# The Transcriptome of the Reference Potato Genome *Solanum tuberosum* Group Phureja Clone DM1-3 516R44

**DOI:** 10.1371/journal.pone.0026801

**Published:** 2011-10-28

**Authors:** Alicia N. Massa, Kevin L. Childs, Haining Lin, Glenn J. Bryan, Giovanni Giuliano, C. Robin Buell

**Affiliations:** 1 Department of Plant Biology, Michigan State University, East Lansing, Michigan, United States of America; 2 James Hutton Institute, Invergowrie, Dundee, United Kingdom; 3 Casaccia Research Center, Italian National Agency for New Technologies, Energy and Sustainable Development, Rome, Italy; University of Arizona, United States of America

## Abstract

Advances in molecular breeding in potato have been limited by its complex biological system, which includes vegetative propagation, autotetraploidy, and extreme heterozygosity. The availability of the potato genome and accompanying gene complement with corresponding gene structure, location, and functional annotation are powerful resources for understanding this complex plant and advancing molecular breeding efforts. Here, we report a reference for the potato transcriptome using 32 tissues and growth conditions from the doubled monoploid *Solanum tuberosum* Group Phureja clone DM1-3 516R44 for which a genome sequence is available. Analysis of greater than 550 million RNA-Seq reads permitted the detection and quantification of expression levels of over 22,000 genes. Hierarchical clustering and principal component analyses captured the biological variability that accounts for gene expression differences among tissues suggesting tissue-specific gene expression, and genes with tissue or condition restricted expression. Using gene co-expression network analysis, we identified 18 gene modules that represent tissue-specific transcriptional networks of major potato organs and developmental stages. This information provides a powerful resource for potato research as well as studies on other members of the Solanaceae family.

## Introduction

Although potato is the third most important food crop after rice and wheat (http://faostat.fao.org), the average yield of potatoes around the world is far below its physiological potential of 120 tons/ha [1]. Advances in potato molecular breeding have been constrained by its complex biological system including vegetative propagation, autotetraploidy, and high levels of heterozygosity [2]. The potato genome [3] and accompanying gene complement are powerful resources for understanding this complex system and advancing molecular breeding efforts in this crop.

The potato gene complement, the corresponding gene structure, chromosome location, and biological function are informative to biologists, breeders, and geneticists. One form of gene annotation is expression profiles, which although correlative, can be used to infer function. Traditional gene expression analyses for potato include Expressed Sequence Tags (ESTs) and microarray-based expression profiles. To date, there are 249,457 potato ESTs in the National Center for Biotechnology Information EST database (dbEST, release 080111; http://www.ncbi.nlm.nih.gov/dbEST/dbEST_summary.html), which have been a valuable resource for gene discovery and expression in several potato genotypes, tissues, and environmental stress responses [4], [5], [6], [7], [8]. Approaches to quantitative gene expression profiling include the development of cDNA and oligonucleotide-based microarrays, for which 26 experiments and 506 assays exist in the National Center for Biotechnology Information Gene Expression Omnibus and the European Bioinformatics Institute ArrayExpress [9], [10]. The Institute for Genomic Research developed potato cDNA microarrays based on ∼12,000 potato clones [11], on which more than 50 studies have been completed including potato development and abiotic/biotic stress responses [11], [12], [13], [14], [15]. An oligonucleotide microarray based on the Agilent microarray platform was used in a series of studies examining tuber growth and metabolism [16]. Although these studies have generated significant amount of data for gene expression analysis, comprehensive characterization of the potato transcriptome has been constrained by limitations in Sanger-based sequencing and array-based methodologies. While Sanger-based EST sequencing is quantitative, cost limitations prevent deep and exhaustive sampling of the transcriptome. Platforms such as the existing potato cDNA and oligonucleotide-based arrays are limited by lack of the full gene complement being interrogated on the platform. Recent advances in high-throughput sequencing technologies have overcome these limitations and whole transcriptome shotgun sequencing, known as RNA-Seq, enables simultaneous analysis of thousands of transcripts for gene discovery and transcript abundance [17]. Moreover, this method provides a comprehensive view of the transcriptome without prior knowledge [18]. To complement the potato genome sequence for the purposes of improving genome annotation and to generate gene expression profiles, members of the Potato Genome Sequencing Consortium (PGSC) generated a large set of next generation transcript sequencing data. Here, we report a reference for the potato transcriptome using the reference accession, the doubled monoploid *Solanum tuberosum* Group Phureja clone DM1-3 516R44 (hereafter referred to as DM).

## Results and Discussion

### Tissues sampled and sequencing metrics

**Figure 1:**
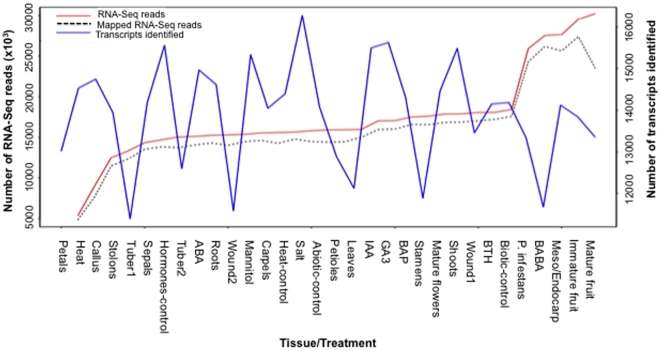
Number of transcripts per tissue as compared to RNA-Seq reads across 32 diverse potato tissues. Values are based on the “Number of RNA-Seq reads”, “Number of mapped reads”, and “Number of transcripts identified” from Tables 1 and 2.

Here, we analyzed gene expression patterns in a set of 32 tissues from DM plants that represent major organs, developmental stages, and stress-related conditions (Tables 1 and 2). We have grouped these tissues into five major classes: Floral (petals, sepals, carpels, stamens, whole flowers), Fruit (mature, immature, inside fruit), Stolon/Tuber (stolons, tuber1, tuber2), Leaf (leaves, petioles), and Other tissues (shoots, callus, roots). Stress conditions included leaves challenged with *Phytophthora infestans*, leaves wounded to mimic herbivory, and the elicitors acibenzolar-s-methyl (BTH) and DL-ß-amino-n-butyric acid (BABA) for biotic stress. For abiotic stress, plants were exposed to drought, salinity, heat, and a panel of four hormones: abscisic acid (ABA), 6-benzylaminopurine (BAP), gibberellic acid (GA_3_), and indole-3-acetic acid (IAA). Overall, this study generated >550 million RNA-Seq reads (35 to 40 base pairs in length). The number of reads per library ranged from 5.4 million in the petal library to 30 million in the mature whole fruit library, while the number of genes that were expressed ranged from 11,394 in tubers to 16,276 in plants treated with NaCl (Tables 1 and 2). We found a weak correlation (−0.14) between the ‘number of transcripts identified’ and the ‘number of RNA-Seq reads’ per library. The minimum and the maximum number of reads both detected a highly similar number of transcripts (Figure 1), suggesting that there was no bias against transcript detection by the depth of sequence coverage in this data set.

**Table 1:** Number of expressed genes and RNA-Seq reads across 16 potato tissues representing different developmental stages.

Tissue	Number of RNA-Seq reads^a^	Number of mapped reads^b^	Number of transcripts identified^c^	Number of high-confidence transcripts^d^
**Floral**				
Carpels	15,601,335	14,277,171	20,097	14,047
Petals	5,380,578	4,869,537	16,489	13,022
Sepals	14,704,187	13,846,242	20,465	14,189
Stamens	17,600,840	16,569,276	18,671	11,887
Whole mature flowers	17,881,237	16,845,116	21,405	14,461
**Fruit**				
Inside of fruit (mesocarp/endocarp)	27,618,354	25,620,388	22,346	14,123
Immature whole fruit	29,511,849	27,370,291	22,155	13,839
Mature whole fruit	30,215,913	23,539,145	20,595	13,359
**Leaf**				
Petioles	15,956,615	14,464,952	19,800	12,870
Leaves	15,983,851	15,028,323	18,992	12,121
**Stolon/Tuber**				
Stolons (above & below ground)	13,336,800	12,318,300	20,027	13,943
Tubers (whole, sample 1)	14,354,215	13,558,765	17,737	11,394
Tubers (whole, sample 2)	15,136,616	14,075,643	19,282	12,595
**Other tissues**				
Callus	12,505,924	11,483,164	20,215	14,744
Roots (*in vitro*)	15,312,261	14,033,241	21,020	14,611
Shoots (*in vitro*)	17,895,194	16,880,654	21,895	15,482
**Overall**	**278,995,769**	**254,780,208**	**26,908**	**21,630**

a Illumina purity filter reads.

b RNA-Seq reads mapped to the DM potato reference genome.

c FPKM value >0.

d After filtering using the two criteria as described above, i.e., a FPKM 95% confidence interval lower boundary greater than zero and FPKM value ≥0.001.

**Table 2:** Number of expressed genes and RNA-Seq reads across 16 libraries from abiotic and biotic treated plants.

Treatment	Number of RNA-Seq reads^a^	Number of mapped reads^b^	Number of transcripts identified^c^	Number of high-confidence transcripts^d^
**Abiotic**				
Control (salt, mannitol, whole plant *in vitro*)	15,922,124	14,421,387	21,078	14,079
Salt (150 µM NaCl, 24 hr)	15,833,384	14,493,134	22,563	16,276
Mannitol (260 µM, 24 hr)	15,555,838	14,636,711	21,800	15,330
ontrol (35°C treatment, whole plant *in vitro*)	15,658,637	14,795,018	21,096	14,391
Heat (24 hr, 35°C)	9,010,310	7,666,373	19,286	14,527
Control (IAA, GA_3_, BAP, ABA, whole plant *in vitro*)	15,054,072	13,752,216	21,829	15,554
IAA (24 hr, 10 µM)	17,038,908	15,969,714	22,132	15,492
GA_3_ (24 hr, 50 µM)	17,061,003	16,021,940	22,301	15,621
BAP (24 hr, 10 µM)	17,509,094	16,604,102	18,289	14,303
ABA (24 hr, 50 µM)	15,258,672	14,322,151	21,343	14,964
**Biotic**				
Control (BTH, BABA, *P. infestans*)	18,430,649	17,585,358	20,663	14,183
BTH - Treated leaves (24 hr/48 hr/72 hr)	18,083,089	17,224,829	20,766	14,149
*Phytophthora infestans* - Infected leaves (24 hr/48 hr/72 hr)	25,893,725	24,285,215	20,857	13,333
BABA - Treated leaves (24 hr/48 hr/72 hr)	27,527,275	26,166,571	19,620	11,675
Leaves (wounding secondary tissue)	15,384,713	14,484,899	18,602	11,585
Leaves (wounding primary tissue)	18,068,314	17,043,054	20,696	13,457
**Overall**	**277,289,807**	**259,472,672**	**24,907**	**19,704**

a Illumina purity filter reads.

b RNA-Seq reads mapped to the DM potato reference genome.

c FPKM value >0.

d After filtering using the two criteria as described above, i.e., a FPKM 95% confidence interval lower boundary greater than zero and FPKM value ≥0.001.

### The DM transcriptome

Transcript abundance is expressed in fragments per kilobase of exon model per million mapped reads (FPKM) as implemented in Cufflinks [19]. This normalized unit allows the comparison both within and between samples. We used two other criteria to filter the expression data sets. First, a transcript was considered expressed if the FPKM 95% confidence interval lower boundary was greater than zero, and second, if the FPKM value was ≥0.001. Based on these criteria, 22,704 high-confidence transcripts were detected in total in these 32 RNA-Seq data sets with 21,630 in the developmental tissue series and 19,704 in the abiotic/biotic stress series (Tables 1 and 2; Table S1). The genome of DM contains 39,031 protein-coding genes [3] and a single transcript was selected to represent each gene model (see Materials and Methods). Thus, the 22,704 transcripts detected here represent nearly 60% of the predicted genes in potato. Eighty-three percent of these transcripts encode proteins with known function. Of the remaining 17%, eight percent had either no match in the UniRef database or lack a Pfam domain with a known function, while nine percent align to an unknown or a hypothetical protein from another species (Table S2). These results indicate that more than half of the transcripts with no known function have sequence homology with other plant proteins, indicating evolutionary conservation and functional significance.

The DM transcriptome data provides a valuable reference for gene expression under normal as well as stress conditions. We identified as many as 20,549 genes expressed in normal tissues of major potato organs. Twenty percent of these (4,184 genes) were exclusive either to floral, fruit, leaf, or stolons/tuber tissues. Similarly, an overall number of 20,390 genes were expressed either in tissue culture, abiotic stress, or biotic stress conditions. Of those, eight percent (1,680 genes) were exclusive to abiotic and/or biotic stress treatments relative to their respective controls. While variation in transcriptome responses are to be expected in other potato species and accessions, the DM abiotic and biotic stress transcriptome profiles provide a baseline assessment of the potato transcriptome that can facilitate further studies in the physiological and biochemical mechanisms of stress responses and adaptation.

Of particular interest are two classes of lineage-specific genes. Comparative analysis of the reference potato DM genome with all available plant genome and transcriptome sequence datasets revealed 2,642 high confidence asterid and 3,372 potato lineage-specific genes [3]. The Asterid-specific set of potato genes encode proteins that lack similarity to any other plant genome or transcriptome except that of another Asterid (see Supplementary Figure 5 in ref [3]). The potato-specific set lack sequence similarity to other plant genome or transcriptome sequence including other Asterids (see Supplementary Figure 5 in ref [3]). Table 3 summarizes the expression of these lineage-specific genes. A total of 779 of the 2,642 Asterid-specific genes (29.5%; Table S6) and 820 of the 3,372 potato-specific genes (24.3%, Table S7) are expressed in at least one tissue. However, only 110 Asterid-specific (14.1%) and 15 potato-specific (1.8%) expressed genes have meaningful functional annotation based on alignments to the UniRef100 database and/or the presence of a Pfam domain. Resistance genes (LRR, late blight resistance, tospovirus resistance) were represented in both classes along with genes encoding systemic acquired resistance protein (Asterid-specific only).

**Table 3:** Expression of Asterid- and potato-specific genes in the DM transcriptome^a^.

	Asterid-specific	Potato-specific
Total genes	2,642	3,372
Expressed genes^b^	779	820
Expressed, unknown function	669	805
Expressed, annotated	110	15
Expressed, disease resistance	9	4
Expressed, Systemic acquired resistance	4	0

a Asterid-specific genes lack sequence similarity to non-Asterid plant genome or transcriptome datasets yet have similarity to at least one Asterid gene. Potato-specific genes lack sequence similarity to any plant genome or transcriptome and thus are restricted to the potato lineage (see Supplementary Figure 5 in ref [3]).

b Expressed were defined as by having a FPKM 95% confidence interval lower boundary greater than zero, and an FPKM value ≥0.001.

### Gene Co-expression Pattern Analyses

To examine the variability in expression levels of constitutively expressed genes, i.e. transcribed in all tissues, we calculated the coefficient of variation (CV = standard deviation/mean) of their FPKM normalized expression counts. Genes with small variation across tissues are thought to perform housekeeping functions and consequently used as reference genes to normalize expression values. When calculated across all 32 samples, the CV ranged from 0.14 to 5.6 (Table S3). In addition to common housekeeping genes such as *glyceraldehyde-3-phosphate dehydrogenase* (PGSC0003DMG400011246, 00015253, 00017433, 00017434), *actin* (PGSC0003DMG400003985, 00018449, 00020244, 02007428), *ubiquitin* (PGSC0003DMG400009125, 00021791, 00023184, 00023462), *tubulin* (PGSC0003DMG400004272, 00014296, 00017954, 00028193, 00029926), and *elongation factor 1-α* (PGSC0003DMG400005728, 00008117, 00019677, 00020772, 00020775, 00023270, 00023272) that have been reported to be stably expressed during biotic and abiotic stress in potato [20], there was a number of genes with high, stable expression levels that could be potentially useful in cross-tissue expression analyses (Table S3).

**Figure 2:**
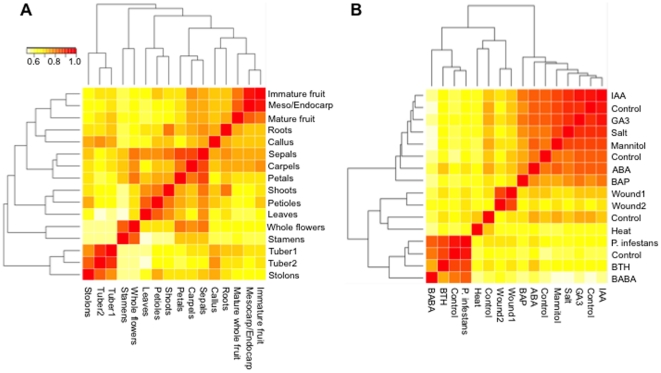
Cluster heat map of gene expression data. The hierarchical clustering was generated using Spearman correlation coefficients of log2-transformed FPKM expression values. A. Correlation among 16 diverse potato organs using 21,630 transcripts. B. Correlation among 16 abiotic and biotic stress-related treatments using 19,704 transcripts. The color scale indicates the degree of correlation (white, low correlation; red, high correlation).

**Figure 3:**
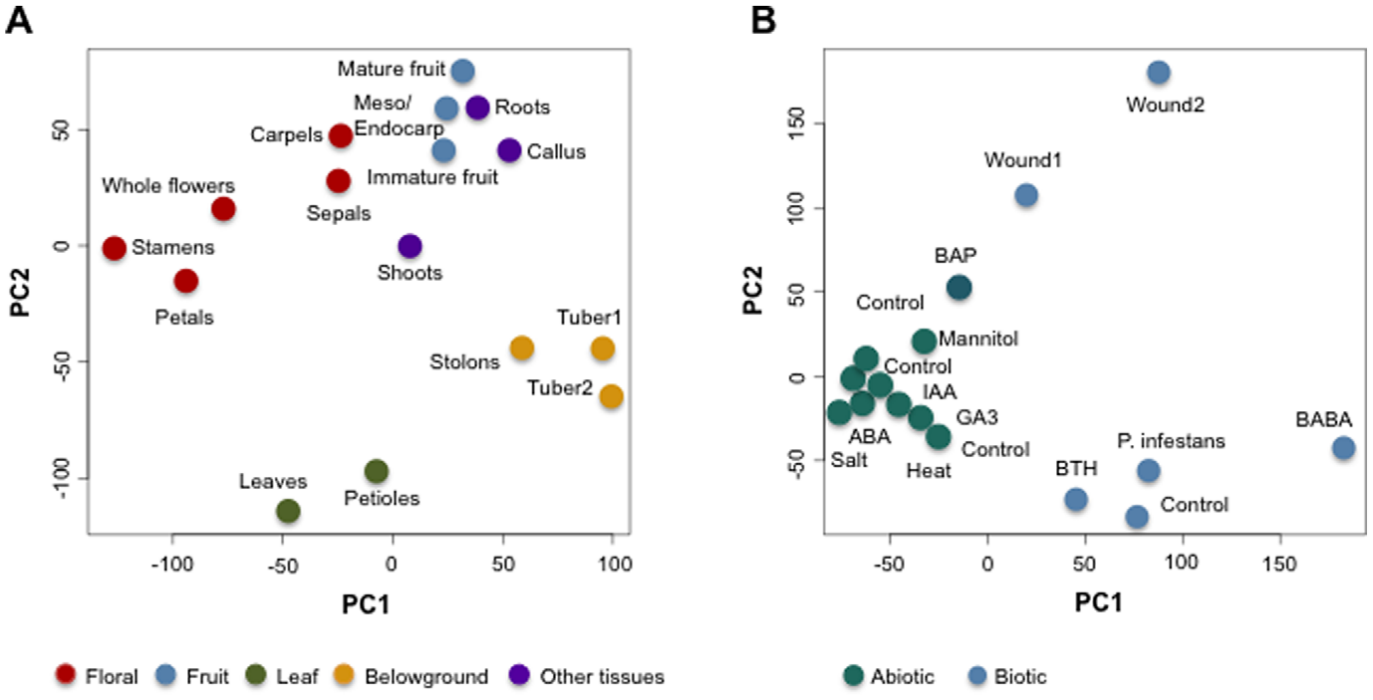
Principal Component Analysis of 16 diverse potato organs based on 21,630 transcripts (A); and 16 abiotic and biotic stress-related treatments based on 19,704 transcripts (B). The plots show the projection of the tissue (A) and treatment (B) samples on the two-dimensional space spanned by the first two principal components. The dots are colored according to tissue types (A) or abiotic/biotic treatments (B).

To better understand the variation of gene expression across all tissue types and stress-related treatments, we performed hierarchical clustering and principal component analyses. Two different RNA-Seq data sets were analyzed: one included 16 different tissue types with 21,630 transcripts; and the other consisted of 16 stress-related treatments with 19,704 transcripts (Figures 2 and 3). The resulting cluster heat maps of log2-transformed FPKM values using the Spearman correlation coefficients clearly differentiated major tissue types as well as biotic and abiotic stresses (Figure 2). Clustering of Floral (sepals, petals, carpels, and stamens, mature whole flowers), Fruit (immature and mature whole fruit), Leaf (leaves, petioles), and Stolon/Tuber tissues (Figure 2A), as well as tissues under abiotic (salt, mannitol, heat, ABA, IAA, GA3) and biotic (late blight, BABA, BAP, BTH, leaf wounding) stresses (Figure 2B) was supported by high bootstrap scores (>90%, 1000 replicates). Similar gene expression patterns were evident when variation among samples was visualized in a reduced-dimension space via the first two principal components (Figure 3). These two principal components together explained only 38% and 43% of the total variation in tissue types and abiotic/biotic stresses, respectively, which may account for overlap between some tissues/treatments. Collectively, these analyses captured the biological variability that accounts for gene expression differences among tissues, and suggest tissue-specific expression of differentially expressed genes as well as genes that are expressed only in a specific tissue type or stress response.

**Figure 4:**
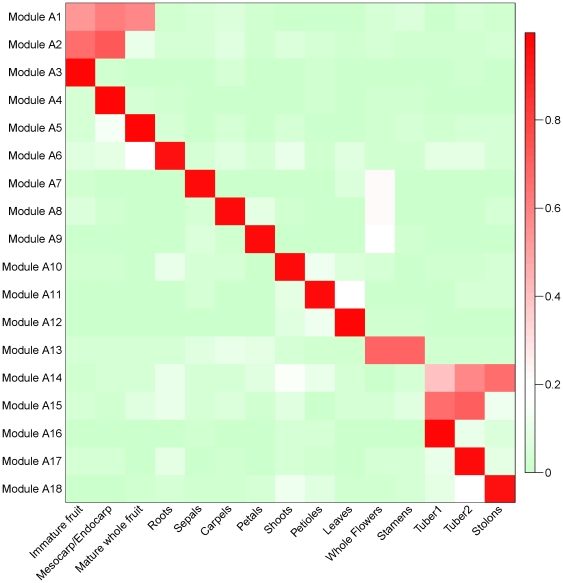
Heat map of the eigengenes representing each gene co-expression module. Rows correspond to eigenegenes for each of the 18 identified gene modules. Columns represent tissue samples. The color scale indicates the relative expression levels of all genes in the module.

**Figure 5:**
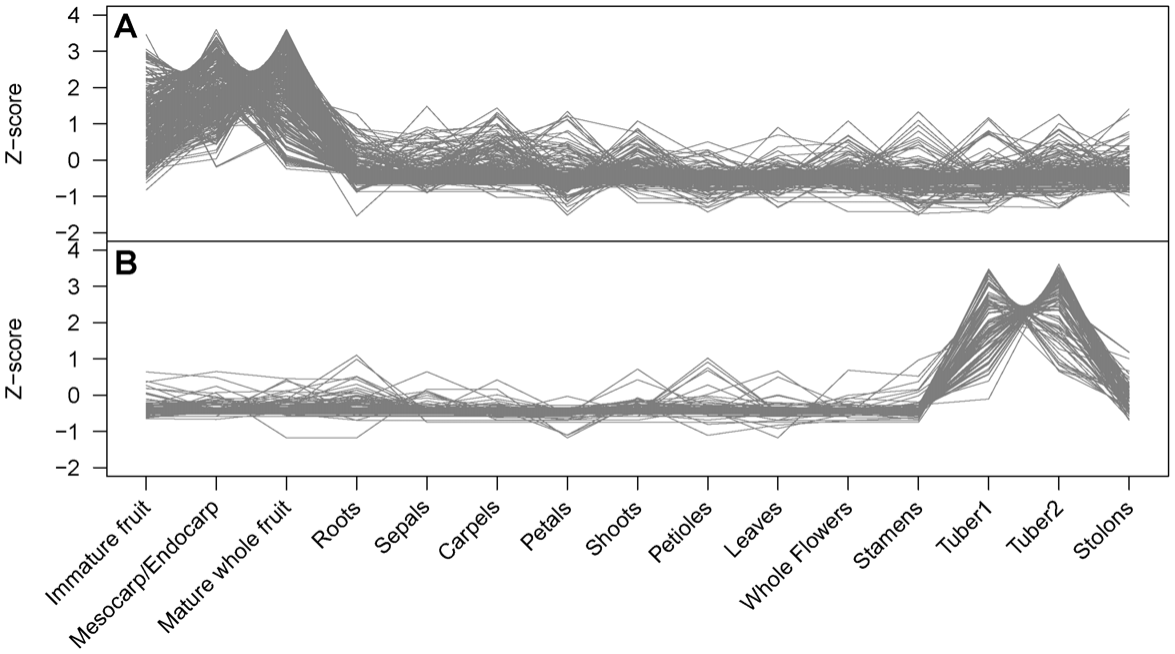
Trend plots of the normalized gene expression values for each gene from two representative modules. A. The 290 genes in module A1 exhibit fruit tissue-specific gene expression. B. The 90 genes in module A15 are most highly expressed in tuber tissues.

A comprehensive identification of highly correlated groups of genes was performed using the Weighted Gene Correlation Network Analysis (WGCNA) [21]. Using 15 tissues from major potato organs and developmental stages, we identified 18 gene co-expression modules containing a total of 5,400 genes (Table S4). Each module represents genes with highly correlated expression profiles, either in a single tissue or in a few developmentally related tissues (Figures 4 and S1). For example, module A1 contains 290 genes that are co-expressed in fruit tissues (“immature fruit”, mesocarp/endocarp”, “mature whole fruit”) (Figure 5A). It included genes involved in fruit development and ripening such as *pectin esterase*, *lipoxygenase*, and *malate synthase* (Table S4). Similarly, module A15 contained 90 genes that are co-expressed in tubers (“tuber1”, “tuber2”), and included starch biosynthesis genes such as *glucose 6-phosphate/phosphate translocator* and storage proteins such as *patatin* (Figure 5B, Table S4).

Our WGCNA analyses identified genes encoding transcription factor-related Pfam domains in all 18 co-expression modules (Table S5). Network modules containing transcription factor genes are of particular importance because these transcription factors may have a role in the regulation of expression of other member genes. Two modules, A2 and A14, were significantly enriched for transcription factors (*P*≤0.001). Module A2, which includes 591 genes co-expressed in fruit development (“Immature fruit”, “Mesocarp/endocarp”), was enriched for proteins containing the *LEAFY COTYLEDON 1* (*LEC1*) (PF00808) and transcriptional factor *B3* (PF02362) Pfam domains. Both the *LEC1* and *B3* domain factors are involved in the regulation of plant embryo development [22], consistent with their expression in fruit containing developing seeds as reported here. Module 14, contained 441 genes co-expressed in tuber tissues (“Tuber1”, “Tuber2”), and was enriched for transcription factors containing the *APETALA* (*AP2*) (PF00847) and WRKY (PF03106) Pfam domains. Some members of the *AP2* gene family, have been previously reported, and also illustrated here, as expressed in swollen stolons and tubers (e.g., GenBank accessions CK720060, DR036046, DR036047).

Overall, analyses of functional assignments of all of the genes within the modules indicate that 30% of the genes in modules have no known function. Examination of the lineage-specific genes revealed that nearly 12% (632 genes) of our module genes are lineage-specific, 289 asterid- and 343 potato-specific (Tables S8, S9). Only a few asterid-specific genes were associated with Pfam domains of known function (e.g., PF07333, PF05938, PF05498, PF04043, Table S8) and these were included in floral (“carpels”, “whole flowers”, “stamens”), tuber, or stolon related co-expression modules (Table S4, Figure 4, modules A8, A13–15). Based on their interaction with known genes, these genes with no meaningful annotation can be used to place these non-annotated genes in a functional context and infer their role in potato development.

In summary, this large dataset of >550 million RNA-Seq reads permitted detection and quantification of expression levels of more than 22,000 genes in the sequenced accession of potato, and provides an overview of the transcriptome of a diverse collection of tissues and growth conditions. Coupled with identification of co-expression modules, these data provide a basis and a powerful resource for future gene expression research in potato and other members of the Solanaceae family.

## Materials and Methods

### Transcriptome profiling

Transcriptome analyses were performed using RNA-Seq data generated by the PGSC described previously [3]. In this data set, transcriptome sequences were generated from 32 DM libraries using RNA-Seq with the Illumina Genome Analyzer II platform (Tables 1 and 2). The 32 DM libraries represent a wide range of developmental tissues/organs as well as abiotic and biotic stress treatments and are described in detail in reference [3] (see Supplementary Material and Table S4). The developmental tissues represent vegetative (leaves, petioles, stolons, tubers sampled twice) and reproductive organs (Floral: carpels, petals, sepals, stamens, whole flowers; Fruit: mesocarp/endocarp, whole immature berries, whole mature berries) from greenhouse-grown plants. Shoots and roots from *in vitro*-grown plants were also included in the developmental series. Callus (10–11 week old) derived from leaves and stems were used to assess transcription in an undifferentiated tissue. The biotic stress conditions (pooled samples at 24 hr, 36 hr, 72 hr) were induced with *Phytophthora infestans* inoculum (Pi isolate US8: Pi02-007) and two chemical inducers, acibenzolar-S-methyl (BTH, 100 µg/ml) and DL-β-amino-n-butyric acid (BABA, 2 mg/ml) using detached leaves. Wounded leaves, primary and secondary, were included to mimic herbivory. The abiotic stress conditions (24 hr treatment of *in vitro* grown whole plants) include heat (35°C), salt (150 mM NaCl) and mannitol (260 µM) treatment. Abscisic acid (ABA, 50 µM), indole-3-acetic acid (IAA, 10 µM), giberellic acid (GA3, 50 µM), and 6 benzylaminopurine (BAP, 10 µM) were used to induce hormone stress responses. Expression levels as previously described in [3] were determined by mapping the RNA-Seq reads to the DM potato reference genome using Tophat [23] and expression levels were determined using Cufflinks [19]. Only representative transcripts, which were chosen by selecting the longest Coding Sequence (CDS) from each gene, were used for the analyses [3]. RNA-Seq reads are available in the NCBI Sequence Read Archive under study number SRA029323.

### Bioinformatic analyses

Functional annotation was performed using a combination of BLASTX searches [24] against the Uniref100 (E-value cutoff of 1e-5) and identification of Pfam domains using InterProScan searches against InterPro [25]. R-statistics (http://www.r-project.org/) were used for hierarchical cluster analysis, cluster dendrograms, and principal component analysis. Domain-enrichment analyses were performed using Fisher's Exact Test as implemented in R (http://cran.r-project.org). Transcription factor genes were identified based on PFAM domains (Table S5).

### Co-expression pattern analyses

Co-expression analysis was performed using WGCNA in order to identify modules of highly correlated genes [21]. CV values were calculated for all genes, and those with a CV less than 0.8 across samples were not included in the WGCNA analyses. Expression values for the remaining genes were then log2 transformed before being processed through the WGCNA R-package [26]. Genes with untransformed FPKM values less than 1 were transformed to zero. For module identification, the WGCNA parameters β and treecut were set to 9 and 0.7, respectively. All other parameters were used with the default values. Eigengenes were calculated for each gene co-expression module in order to visualize the gene expression patterns for each module. Eigengenes are the first principal component of principal component analysis of the normalized expression values of all genes in a module, and they represent the average normalized gene expression for a module [27].

## Supporting Information

Figure S1 **Trend plots of the normalized gene expression values for each gene from eighteen identified gene coexpression modules.** Modules consisting of genes with specific in various tissues: A1. mesocarp/pericarp tissue and mature fruit, A2. immature fruit and mesocarp/pericarp tissue, A3. immature fruit, A4. mesocarp/pericarp tissue, A5. mature fruit, A6. roots, A7. sepals, A8. carpels, A9. petals, A10. shoots, A11. petioles, A12. leaves, A13. whole flowers and stamens, A14. tubers and stolons, A15. Tubers (sample 1 and 2), A16. tubers (sample 1), A17. tubers (sample 2) and A18. stolons.
(PDF)

Table S1 **List of high-confidence transcripts detected in 32 tissues with corresponding gene and peptide IDs.**
(XLS)

Table S2 **List of high-confidence transcripts with corresponding putative function, as determined by BLASTX searches against UniRef100 (E-value cutoff of 1e-5), and Pfam domains.**
(XLS)

Table S3 **List of constitutively expressed genes, their FPKM values (columns 2–33), coefficient of variation (CV = Standard deviation/Mean), putative function, and Pfam domains.**
(XLS)

Table S4 **List of modules (A1 to A18) with their corresponding gene ID, putative function as determined by BLASTX searches against UniRef100 (E-value cutoff of 1e-5), and Pfam domain/s.**
(XLS)

Table S5 **List of modules with their corresponding peptide ID and transcription factor-related Pfam domains.**
(XLS)

Table S6 **List of expressed asterid-specific genes with functional annotation.**
(XLSX)

Table S7 **List of expressed potato-specific genes with functional annotation.**
(XLSX)

Table S8 **List of asterid-specific genes identified in tissue-related co-expression modules.**
(XLSX)

Table S9 **List of potato-specific genes identified in tissue-related co-expression modules.**
(XLSX)
