# Supplementary figures and images for: The Transcriptome of the Reference Potato Genome Solanum tuberosum Group Phureja Clone DM1-3 516R44
Source: PLoS One. 2011 Oct 28;6(10):e26801. doi: 10.1371/journal.pone.0026801 (PMC3203163; doi:10.1371/journal.pone.0026801)

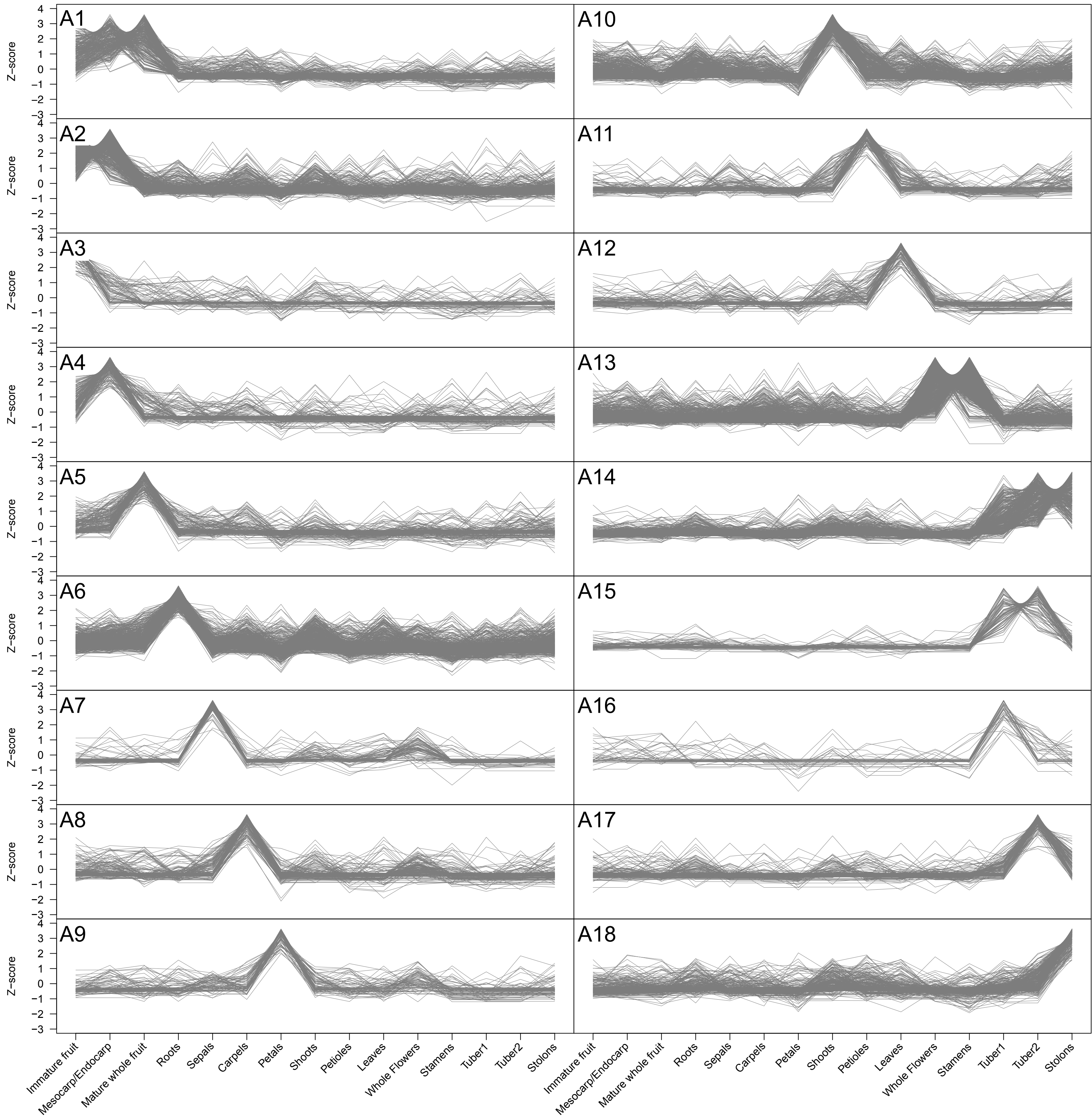

Supplement: Figure S1 — Trend plots of the normalized gene expression values for each gene from eighteen identified gene coexpression modules. Modules consisting of genes with specific in various tissues: A1. mesocarp/pericarp tissue and mature fruit, A2. immature fruit and mesocarp/pericarp tissue, A3. immature fruit, A4. mesocarp/pericarp tissue, A5. mature fruit, A6. roots, A7. sepals, A8. carpels, A9. petals, A10. shoots, A11. petioles, A12. leaves, A13. whole flowers and stamens, A14. tubers and stolons, A15. Tubers (sample 1 and 2), A16. tubers (sample 1), A17. tubers (sample 2) and A18. stolons. (PDF) [file pone.0026801.s001.pdf]
